# Regulation of ROCK1 via Notch1 during breast cancer cell migration into dense matrices

**DOI:** 10.1186/1471-2121-13-12

**Published:** 2012-05-14

**Authors:** Vanisri Raviraj, Sandra Fok, Jifei Zhao, Hsin-Ya Chien, J Guy Lyons, Erik W Thompson, Lilian Soon

**Affiliations:** 1 Australian Centre for Microscopy and Microanalysis (ACMM), AMMRF, The University of Sydney, Sydney, NSW 2006, Australia; 2 Dermatology, Central Clinical School, The University of Sydney, Sydney, NSW 2006, Australia; 3 Invasion and Metastasis Unit, St. Vincent’s Institute of Medical Research, Melbourne, Australia; 4 University of Melbourne Department of Surgery, St Vincent’s Hospital, Melbourne, Australia; 5 ACMM, Madsen Building F09, Room 243, The University of Sydney, Sydney, NSW 2006, Australia

**Keywords:** Breast cancer, High-density matrix, Cancer cell migration, ROCK expression, Histone deacetylase inhibitors

## Abstract

**Background:**

The behaviour of tumour cells depends on factors such as genetics and the tumour microenvironment. The latter plays a crucial role in normal mammary gland development and also in breast cancer initiation and progression. Breast cancer tissues tend to be highly desmoplastic and dense matrix as a pre-existing condition poses one of the highest risk factors for cancer development. However, matrix influence on tumour cell gene expression and behaviour such as cell migration is not fully elucidated.

**Results:**

We generated high-density (HD) matrices that mimicked tumour collagen content of 20 mg/cm^3^ that were ~14-fold stiffer than low-density (LD) matrix of 1 mg/cm^3^. Live-cell imaging showed breast cancer cells utilizing cytoplasmic streaming and cell body contractility for migration within HD matrix. Cell migration was blocked in the presence of both the ROCK inhibitor, Y-27632, and the MMP inhibitor, GM6001, but not by the drugs individually. This suggests roles for ROCK1 and MMP in cell migration are complicated by compensatory mechanisms. ROCK1 expression and protein activity, were significantly upregulated in HD matrix but these were blocked by treatment with a histone deacetylase (HDAC) inhibitor, MS-275. In HD matrix, the inhibition of ROCK1 by MS-275 was indirect and relied upon protein synthesis and Notch1. Inhibition of Notch1 using pooled siRNA or DAPT abrogated the inhibition of ROCK1 by MS-275.

**Conclusion:**

Increased matrix density elevates ROCK1 activity, which aids in cell migration via cell contractility. The upregulation of ROCK1 is epigenetically regulated in an indirect manner involving the repression of Notch1. This is demonstrated from inhibition of HDACs by MS-275, which caused an upregulation of Notch1 levels leading to blockade of ROCK1 expression.

## Background

Cancer is a complex disease and is strongly influenced by a number of factors including genetics, epigenetics, behavioural aspects and the environment. At the cellular level, these factors impact on cell signalling leading to uncontrolled proliferation and cell migration with adverse consequences in the formation of tumours and metastases. The stroma is known to regulate mammary gland development [1] and under some circumstances, also promote breast cancer [2]. Stromal contents include fibroblasts [3,4], immune cells [5,6], adipocytes [7], and extracellular matrix (ECM), which can regulate the survival, proliferation and invasion of tumour cells.

Breast cancers have a high stromal content, which is characterized by activation of fibroblasts (from non-proliferative fibroblasts to proliferative myofibroblasts), increased vascularisation, increased deposition of stromal collagen, and cross-linking and reorientation of ECM [8,9]. Increased deposition of stromal collagen or “desmoplasia” is associated with enhanced matrix stiffness. Desmoplasia can promote the proliferation of normal and transformed cells and increase cell invasion and metastasis [10,11]. Furthermore, high mammographically dense tissue (HMT) as a pre-existing condition poses one of the highest risk factor for breast cancer development [12]. The predominant component of high mammographically dense tissue is connective tissue consisting mostly of collagen [13]. Provenzano et al., [14] studied mice with a mutation in the α1(I) chain of type 1 collagen that dramatically reduces collagen proteolysis. Mammary glands of these mice exhibit increased collagen deposition and show signs of hyperplasia such as irregular epithelial boundaries.

Single tumour cell migration in three-dimensional (3D) matrices can be classified into broad categories known as amoeboid and mesenchymal migration. Single tumour cell migration in three-dimensional (3D) matrices can be classified into broad categories known as amoeboid and mesenchymal migration. Mesenchymal migration is characterized by elongated spindle-like cell morphology and requires integrin-mediated matrix-focal adhesion interactions, cortical F-actin, stress fibres formation, and expression of proteases [15]. Unlike mesenchymal migration, amoeboid cells do not require proteolysis or integrins for migration [16,17]. Amoeboid migration refers to movement of rounded or ellipsoid cells, which has no mature focal adhesions and stress fibres [18]. Cell movement is driven by actin polymerization and rapid expansion and contraction of the cell body that allows the cells to squeeze through pores in the ECM [16]. Amoeboid- and mesenchymal-like cells might utilize proteolysis for migration depending on the density [19], the presence of cross-links [20] and the fibrillar or non-fibrillar nature of the matrices [21]. In the lower spectrum of matrix densities, amoeboid-like tumour cells exhibit non-proteolytic migration by Rho-associated coiled-coil forming kinase (ROCK)-associated contractility or protrusion-led mechanisms [22,23]. Protrusion-led migration occurs independently of integrins and it is driven by protrusions [23]. This can occur in low-density collagen matrices where contraction-inhibited dendritic cells are able to reach the same instantaneous velocity peak as control cells mainly through pushing forces generated by actin polymerisation during protrusion formation [23].

Increased expression of RhoA or RhoC GTPAses and/or their ROCK1/2 effectors has been reported in several metastatic cancers [24], and they play important roles in tumour progression and invasion [25,26]. These molecules also partake in mechanotransduction of signals in response to external tensional stimuli [11,27].

This work investigates the role of dense collagen matrices that resemble the matrix densities of mammary breast cancer tissues in regulating the migration of tumour cells. MTLn3 rat mammary carcinoma cells were observed to maneuver between collagen fibrils during migration into dense matrices utilizing cell contractility. These cells also have significantly higher ROCK1 activity levels in high-density (HD) compared to low-density (LD) matrices, indicating matrix-dependent regulation. ROCK1 levels and activity were sensitive to HDAC inhibition by MS-275, which was abrogated when Notch1 was blocked. Inhibition of ROCK1 and metalloproteases by themselves had no effect on cell migration indicating alternation of invasion strategies. However, in the presence of both inhibitors, cell migration was significantly blocked.

## Results

### Preparing *in vitro* collagen matrices with similar collagen content and organization to high mammographically-dense tissues (HMT)

**Figure 1:**
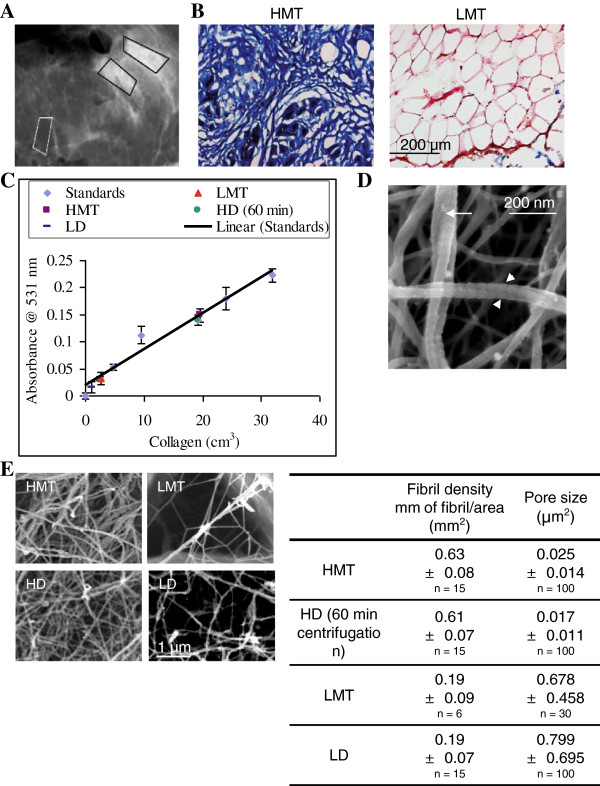
** Preparation of HD collagen gels resembling desmoplastic matrices.A**, High-density mammographic tissue (HMT) and low-density mammographic tissue (LMT) denoted by light and dark radiographic areas respectively, were macrodissected from DCIS mammary tissue and processed. **B**, Masson’s Trichrome staining showed abundant collagen fibrils in HMT tissue (blue stain) and mostly adipose cells in LMT tissue. **C**, *In vitro* HD matrix was prepared by centrifugation to concentrate solubilised collagen followed by polymerisation using vaporised NH_4_OH. HD matrix along with HMT and LMT samples were stained with picrosirius red and assayed for dye binding at 531 nm wavelength of light. The collagen concentration of HMT (■), LMT tissues (▴) and HD collagen matrix (●) were extrapolated from standards of known collagen concentrations (♦). After 60 min of centrifugation, HD collagen measured 19.16 ± 0.74 mg/cm^3^, which closely correlated with HMT samples of 19.59 ± 2.91 mg/cm^3^ collagen. **D**, SEM of HMT tissues and HD matrix shows that collagen is organized as networks of dense fibrils. Field emission SEM demonstrates that HD collagen consists of small ~30 nm collagen fibrils that form helical coils of larger 200 nm fibrils (arrows). Collagen D-spacing of ~50–60 nm is visible along the lengths of individual collagen fibrils and are aligned relative to adjacent coiled fibrils (arrowheads). **E**, the fibril density and pore sizes of HD (60 min centrifugation) and LD matrices closely mimicked HMT and LMT counterparts of tumour tissue (n = no. areas sampled). Experiments were repeated three times. Bars indicate standard deviation from triplicate samples. Scale bars represent 200 μm in B and 1 μm in D.

**Figure 2:**
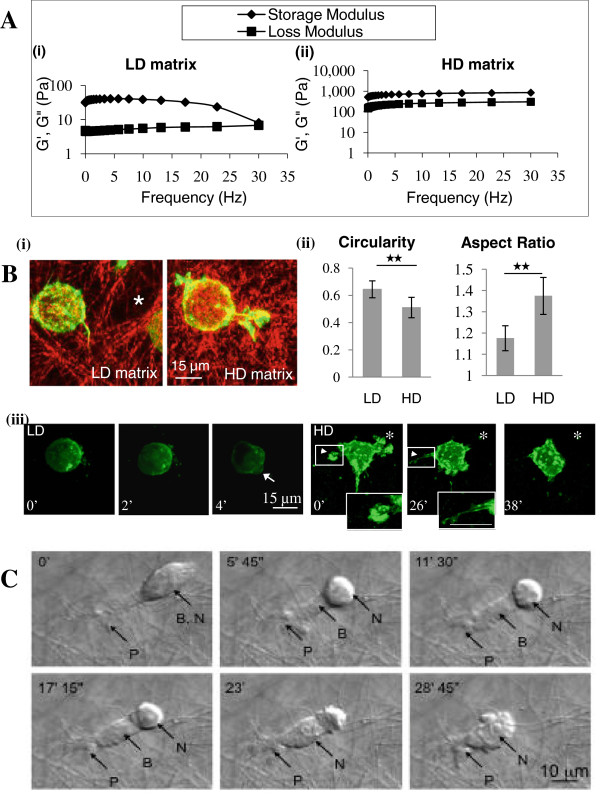
** Matrix properties and cell migration.** Tumour cells were grown in low-density (LD) matrix of 1 mg/cm^3^ or high-density (HD) matrix of 20 mg/cm^3^. **A**, The storage modulus (G’, ♦) and the loss modulus (G”, ■) of matrices were graphed against oscillation frequency sweeping from 0.01 to 30 Hz. As the rate of deformation increases, the storage modulus and loss modulus values converge at high deformation rates in LD but not HD matrix reflecting higher viscoelastic properties in the latter. **B**, (i), Merged reflection and confocal images of collagen and paxillin-Alexa 568-labelled tumour cells demonstrate larger interfibrillar spaces (*) in LD matrix. (ii) Morphometric indicators show that tumour cells are more rounded in shape in LD compared to HD matrices (n = 15). (iii), Live-cell confocal microscopy of GFP-actin transfected MTLn3 cells. Cells in LD matrix appear rounded and form blebs during migration (arrow). In HD matrix, protrusions are extended via ruffling (arrow heads) and terminate as fine filopodia (inset images). Asterisk indicates the initial cell position. **C**, Live-cell DIC microscopy shows a typical migration pattern of a tumour cell through HD matrix. At time 0’, a cell protrusion (P) has breached the fibrous mass. At 5’ 45”, the cell body (B) has partially extended into the matrix via cytoplasmic propulsion leaving behind the rounded, tall nucleus (N). In later frames, contraction of the cell body facilitated squeezing of the nucleus past obstructing matrix fibrils, completing the migration cycle by 28’ 45”.

Regions of low or high mammographic density in prophylactic invasive ductal carcinoma (IDC) tissues were macrodissected and processed for imaging and quantitative analyses (Figure 1A). Masson’s Trichrome staining showed that HMT regions contained mostly collagen (90%) with isolated clusters of glandular cells whereas low mammographically-dense tissues (LMT) consisted mainly of adipose cells with little presence of collagen (Figure 1B). Collagen concentrations were estimated using picrosirius red to stain collagen and measuring dye uptake at 531 nm absorbance wavelength. Compared against known standards, the collagen content in LMT and HMT were measured to be 2.65 ± 1.60 and 19.59 ± 2.91 mg/cm^3^, respectively (Figure 1C). High-density (HD) collagen matrix was prepared by centrifugation to increase collagen concentrations and polymerisation using vaporised NH_4_OH. A centrifugation time of 60 min was found to be suitable for preparing HD matrices at 19.16 ± 0.74 mg/cm^3^, similar to that for HMT extracts (Figure 1C). The fibril densities were comparable between HMT tissue and HD matrix measuring 0.63 ± 0.08 and 0.61 ± 0.07 mm of fibril/mm^2^, respectively (Figure 1D). Similarly, in LMT tissue, the density 0.19 ± 0.09, was similar to that of LD matrix which measured 0.19 ± 0.07 mm of fibril/mm^2^. Pore sizes between tumour tissue and *in vitro* matrices are also comparable with HMT and HD pore sizes measuring 0.025 ± 0.014 and 0.017 ± 0.011, respectively, while those of LMT and LD measure 0.678 ± 0.458 and 0.799 ± 0.695, respectively. The collagen fibril size of the HD matrix was very similar to HMT tissue, with 62% of the fibrils lying within 125–225 nm for both matrices (data not shown). Furthermore, the matrices resembled the collagen nanostructures and fibrillar networks found in native tissues [28,29], evident from the presence of ~63 nm D spacings and helical fibril conformations (Figure 1E). To understand the relationship between cell migration and matrix density, we compared the properties of low-density (LD) (1 mg/cm^3^) and high-density (HD) (20 mg/cm^3^) collagen matrices. LD matrix contained larger pore spaces and was less viscoelastic compared to HD matrices (Figure 2A, B).

### Physical matrix properties, cell migration, and gene expression

Tumour cells of epithelial origins migrate away from the primary tumour by first breaching the basement membrane, which has low values of Young’s modulus or low resistance to elastic deformation. Paszek and Weaver, 2004 [10], measured this to be 175 ± 37 Pa for reconstituted basement membrane, which is similar to the elastic modulus of collagen matrices at 1–2 cm^3^. Our measurements of 1 cm^3^ collagen matrices also fall on the low end at <100 Pa. By contrast, HD matrix is approximately 10-fold stiffer compared to LD matrix. This suggests that HD matrix recovered more easily from deformation while the latter was more susceptible to deformation forces (Figure 2A). Therefore, tumour cells essentially cross from a low collagen content and “malleable” milieu of the basement membrane and into highly dense and rigid collagen matrices (Figure 1). It was also recently shown that tumour cells are attracted to regions of high matrix stiffness, a mechanism known as durotaxis [30]. The present model is designed to study how tumour cells enter HD collagen matrix similar in density to tumour matrices. Tumour cells are seeded on top of the HD matrix, which mimics the breaching of tumour cells from a region of low or negligible stromal density into highly dense tumour stroma. Seventy-two hours after seeding, 89% (n = 4 experiments) and 100% (n = 2 experiments) of cells have invaded into LD and HD matrices, respectively. Cell shape was determined from measurements of Circularity and Aspect Ratio. Circularity ratios approaching 1.0 represent a perfect circle while Aspect Ratio is the length of the major axis divided by the minor axis. Cells in LD matrix have larger Circularity values and lower Aspect Ratio values than those in HD matrix suggesting a rounder cell shape (Figure 2B). Live-cell confocal microscopy shows that in LD matrix, the cell appears rounded and during migration, forms membrane blebs due to contractility of the cortex. In HD matrix, a very different morphology is apparent where a large protrusion forms through a series of membrane ruffling and extension events leading to the generation of fine filopodia distally (Figure 2B). In HD matrix, the protrusive cell front leads the migration process. The nucleus, being a tall and rigid structure, required a long period to move past barricading matrix fibrils. This was assisted by cytoplasmic streaming and cell contractility that forced the cell body into the HD matrix (Figure 2C; Additional file 1: Movie S1).

### The expression of ROCK1 transcript and ROCK protein activity are increased in high-density matrix

**Figure 3:**
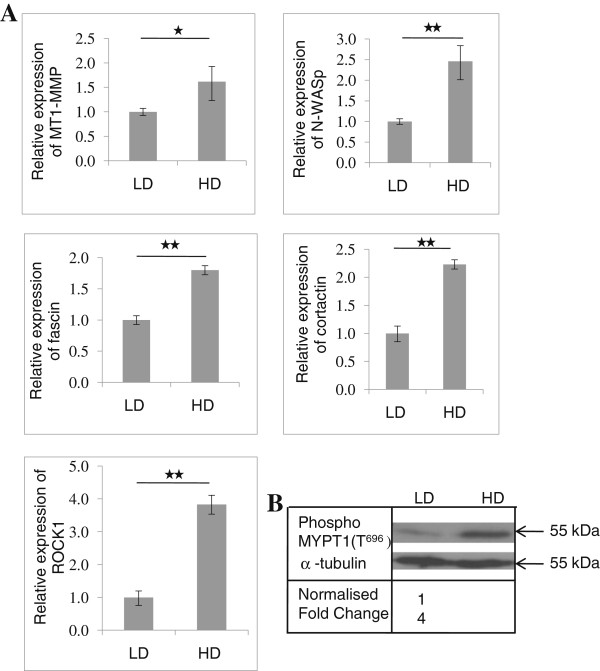
** Effect of matrix densities on the expression of invasion- related genes.** MTLn3, rat breast cancer cells were cultured in LD (1 mg/cm^3^) and HD (20 mg/cm^3^) collagen matrices and the cells were allowed to invade in the matrices for 72 h and the total RNA was used to quantify the gene expression levels by RT- qPCR. Gene expression levels were normalized to GAPDH expression and relative expression was calculated using the ΔΔC_t_ method. **A**, The expression of MT1-MMP, N-WASp, fascin, cortactin and ROCK were significantly increased in HD matrix. **B**, ROCK activity assay and western blotting showed that HD matrix significantly increased the ROCK activity 4-folds compared to LD matrix. The experiment was repeated three times. Bars indicate the standard error from three biological triplicates. Student’s *t*-test show significant difference at either * *p* < 0.05 or ** *p* < 0.01.

To investigate whether matrix densities might alter the expression of invasion-related genes, we performed quantitative RT-PCR using total RNA from cells migrating in LD and HD matrices. HD collagen matrix significantly increased MT1-MMP, N-WASp, fascin, cortactin and ROCK1 (Figure 3A). To further investigate how matrix density might affect ROCK, its expression and protein activity were quantified. Quantitative PCR results revealed up to 4 fold higher expression of ROCK1 transcript in HD matrix compared to LD matrix. ROCK kinase activity assay was performed using the recombinant myosin phosphatase targeting subunit 1 (MYPT1) as a ROCK substrate and anti-phospho-MYPT1 (Thr^696^) as the labeling antibody. ROCK inactivates myosin phosphates through specific phosphorylation of myosin phosphatase target subunit1 at Thr^696^ [MYPT1 (Thr^696^)]. ROCK activity was also significantly increased in HD suggesting that MTLn3 cancer cells use more active ROCK to invade through denser matrix (Figure 3B).

### ROCK inhibition suppressed cell invasion in a context-dependent manner

**Figure 4:**
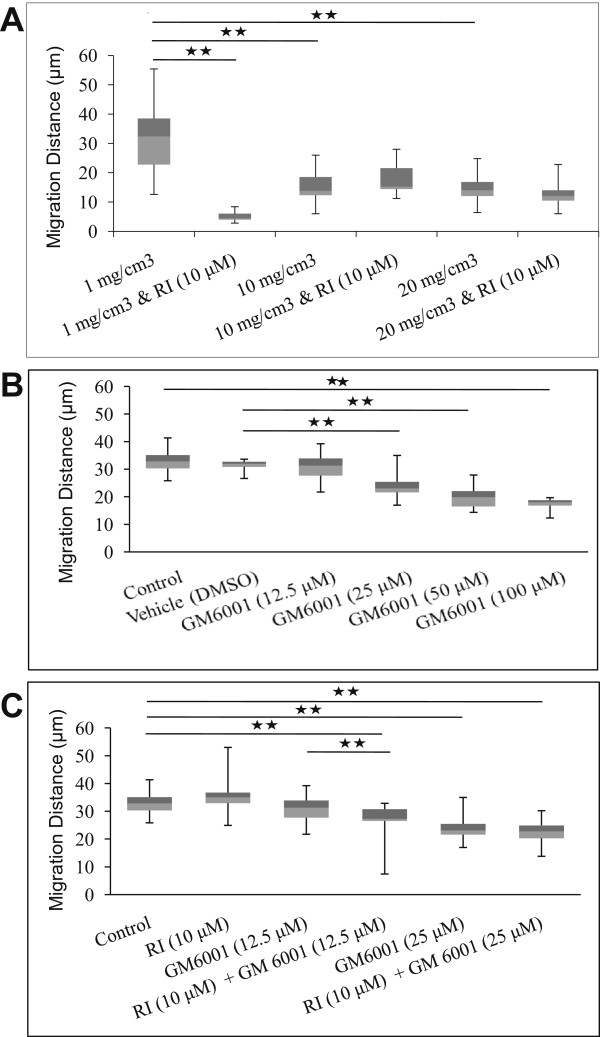
** Migration of MTLn3 carcinoma cells in HD matrix.** Tumour cells were seeded on matrices for 24 h and treated with vehicle, the rock inhibitor (RI), Y-27632, and the MMP inhibitor, GM6001, and allowed to migrate for a further 48 h. The cells were stained with phalloidin-Alexa488 and imaged by confocal and reflection microscopies to capture cells and collagen, respectively. The image stacks were reconstructed in the x-z plane to measure the depth of cell migration. **A**, The RI blocked cell migration in 1 mg/cm^3^ matrix but had no significant effects on cell migration in higher density matrices of 10 and 20 mg/cm^3^. **B**, GM6001 significantly inhibited cells migration at higher concentrations but had no effect at the lowest concentration of 12.5 μm. C, Cell migration was significantly inhibited in the presence of both RI (10 μm) and low concentrations of GM6001 (12.5 μm) compared to vehicle-treated cells alone. Grey and white boxes show the upper and lower quartiles, respectively. Upper bars represent the maximum and lower bars, the minimum distance travelled by cells. Each experiment was repeated at least twice. 1-way ANOVA followed by post-hoc Tukey’s test indicating significant difference at * *p* < 0.05 and ** *p* < 0.01.

The methodology we apply for studying cell migration is consistent with the above observations. Here, tumour cells are seeded on top of matrices, and allowed to migrate for several days simulating possible scenarios *in vivo* where cells might migrate through matrices with densities that are from close to zero to as high 20 mg/cm^3^. Projections of the x-z plane of confocal microscope indicate that the tumour cells migrate deeper into the matrix over time. By 72 h, imaging data confirmed that most cells have become completely submerged into the matrices (not shown). In order to determine how inhibiting ROCK might affect the migration of tumour cells, 48 h assays were performed in the presence or absence of the rock inhibitor, Y-27632. Box and whiskers plot indicate that 50% of cells lying between the upper and lower quartiles are migrating distances of between 23 and 40 μm in LD matrix and 12–18 μm in denser matrices of 10–20 mg/cm^3^ (Figure 4A). In LD matrix, the presence of the ROCK inhibitor reduced the values to 4–6 μm but there were no significant effects on cell migration in the higher density matrices (Figure 4A). It is possible that the tumour cells are adaptable to the inhibitor, switching to either protrusion- and/or protease-lead migration modes, masking the effects of Y-27632. To test this, tumour cells were incubated with Y-27632 as well as GM6001, the wide spectrum metalloproteinase inhibitor. The MMP inhibitor, GM6001, has been utilized over a range of concentrations from 10 to 50 μM [31,32]. At a critical concentration of the GM6001 inhibitor (12.5 μM), addition of Y-27632 significantly blocked cell migration whereas the presence of either inhibitor alone had no effect (Figure 4B, C).

### MS-275 downregulated the protein expression and kinase activity of ROCK1 in HD matrices

**Figure 5:**
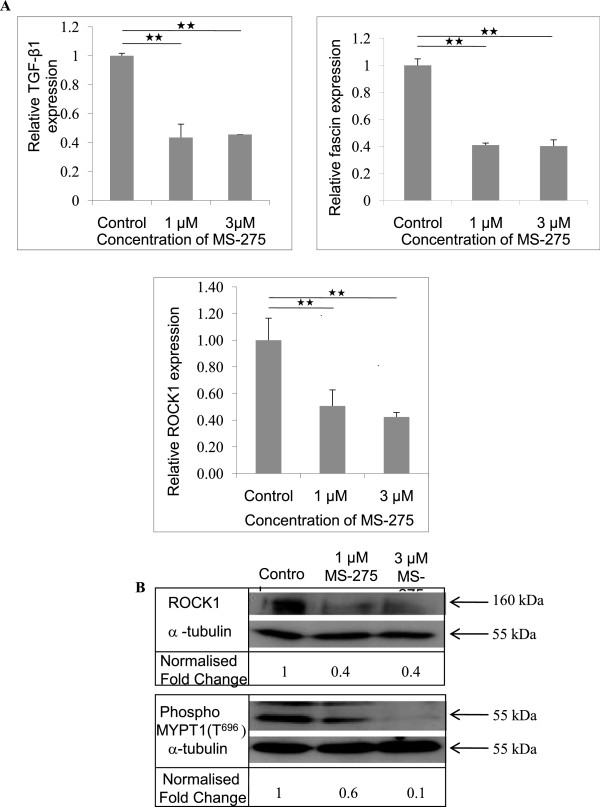
** MS-275 reduced the expression of invasive genes including ROCK1 in HD matrices.** The HDAC inhibitor, MS-275, was used to treat cells 24 h after seeding in HD matrix and the cells were harvested 48 h after treatment for either qPCR or for ROCK activity assay. **A**, Real-time PCR results showed a significant decrease in the expression of TGF-β1, fascin and ROCK1 following treatment with MS-275. Relative expression was calculated using ΔΔC_t_ method. **B**, Western blotting and activity assay for ROCK1 demonstrated, the reduction in protein level and its activity level following treatment of MS-275. Ratio measurements were obtained following normalisation with α–tubulin. Bars indicate standard error from two biological replicates experiments and experiments were repeated three times. 1-way ANOVA followed by post-hoc Tukey’s test indicating significant difference at ** *p* < 0.01.

The cellular microenvironment governing cell adhesion was previously shown to regulate ROCK expression via epigenetic means [33,34]. We hypothesise that matrix density might also modulate ROCK in similar ways. To test this, we used the histone deacetylase inhibitors, MS-275 and valproic acid (VPA), to investigate whether histone modification might be responsible for ROCK activation in HD matrix. There are several naturally occurring and synthetic HDAC inhibitors including trichostatin A (TSA), suberoylanilide hydroxamic acid (SAHA), MS-275 and VPA. MS-275 is an orally active, synthetic HDAC inhibitor that selectively inhibits class 1 HDACs such as HDAC1 and HDAC3 [35]. This benzamide derivate has better physicochemical (bio availability and stability) properties and less toxicity than trichostatin (TSA) [36,37]. It has been tested in over 60 human cancer cell lines, a variety of human tumour xenograft models and in patients with advanced acute leukemia, advanced solid tumours or lymphoma [38,39]. Srivastava et al., 2010 reported that treatment of MS-275 in a breast cancer mouse model (MDA-MB-468 cells), induced growth arrest, apoptosis, and inhibited angiogenesis, migration and metastasis [39]. VPA is an established and well-tolerated drug for epilepsy. It inhibits class I HDACs and also shows anti-tumour activity in a variety of human cancer cell lines including estrogen-sensitive and estrogen- insensitive breast cancer cell lines [40,41]. It is less toxic compared to TSA [42] and is in phase II and III clinical trials for many human cancers [43]. MS-275 and VPA are selected for this study for their functional similarity and low toxicity. In HD matrix, the expression of genes associated with invasion such as TGF-β1, ROCK1 and fascin were shown to be significantly downregulated by MS-275 (Figure 5A). VPA similarly significantly downregulated ROCK1 at the transcript level ( Additional file 2: Figure S1). In addition, Western blotting demonstrated that the amount of ROCK1 protein was significantly reduced by 60% in the presence of 1 and 3 μM of MS-275 (Figure 5B). Similarly, a kinase activity assay followed by immunoblotting showed that ROCK kinase activity was significantly inhibited by treatment with 1 and 3 μM of the MS-275 inhibitor, by 40% and 90%, respectively (Figure 5B).

### Blocking protein synthesis abrogated the downregulation of ROCK1 by MS-275 in HD matrix

**Figure 6:**
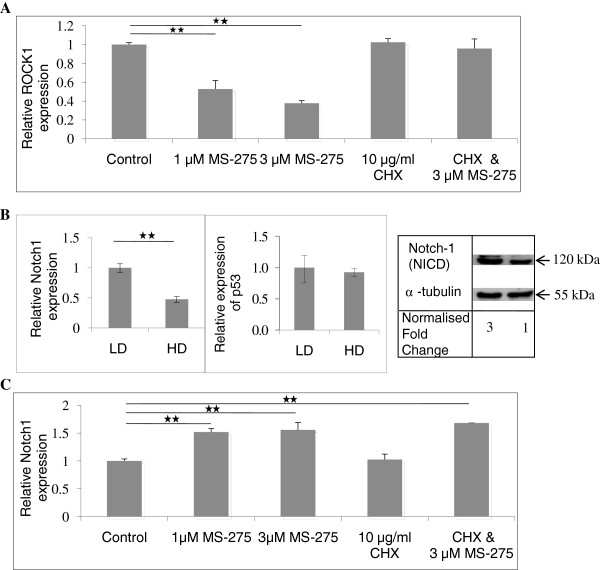
** Reduction of ROCK1 by MS-275 is dependant on protein expression.** MTLn3 breast cancer cells were allowed to invade into HD matrices for 24 h before treatment with 1 μM and 3 μM MS-275, 10 μg/ml of cycloheximide (CHX) and both CHX and MS-275 for a further 24 h or cells were grown on LD and HD matrix for 72 h to total RNA and protein extraction. **A**, Real-time PCR results showed that MS-275 alone decreased ROCK1 expression but this was reversed in the presence of CHX. **B**, HD significantly suppressed Notch-1 expression at both mRNA and protein level compared to LD matrix and there was no change in p53 expression. **C**, MS-275 upregulated Notch1 levels and this was unaffected by CHX. Bars indicate standard error from two biological samples and experiments were performed two times. 1-way ANOVA followed by post-hoc Tukey’s test indicating significant difference at either * *p* < 0.05 or ** *p* < 0.01.

MS-275 downregulated ROCK1 expression at the mRNA and protein levels as well as the activity of ROCK1. We next asked whether the inhibition of ROCK1 is a direct effect of MS-275. Cycloheximide (CHX) is a well-known protein inhibitor that blocks protein synthesis *in vivo* and *in vitro*. CHX interferes the translocation of amino acids to ribosome by affecting the ribosomal donor site, thereby blocking translational initiation and elongation [44]. The use of CHX at 10 μg/ml together with MS-275 abrogated the effects of MS-275 alone, leading to ROCK1 levels that matched those of the control untreated sample (Figure 6A). Therefore, *de novo* protein synthesis is needed for the downregulation of ROCK1 by MS-275.

### HD matrix reduced Notch1 expression that was abrogated by MS-275

**Figure 7:**
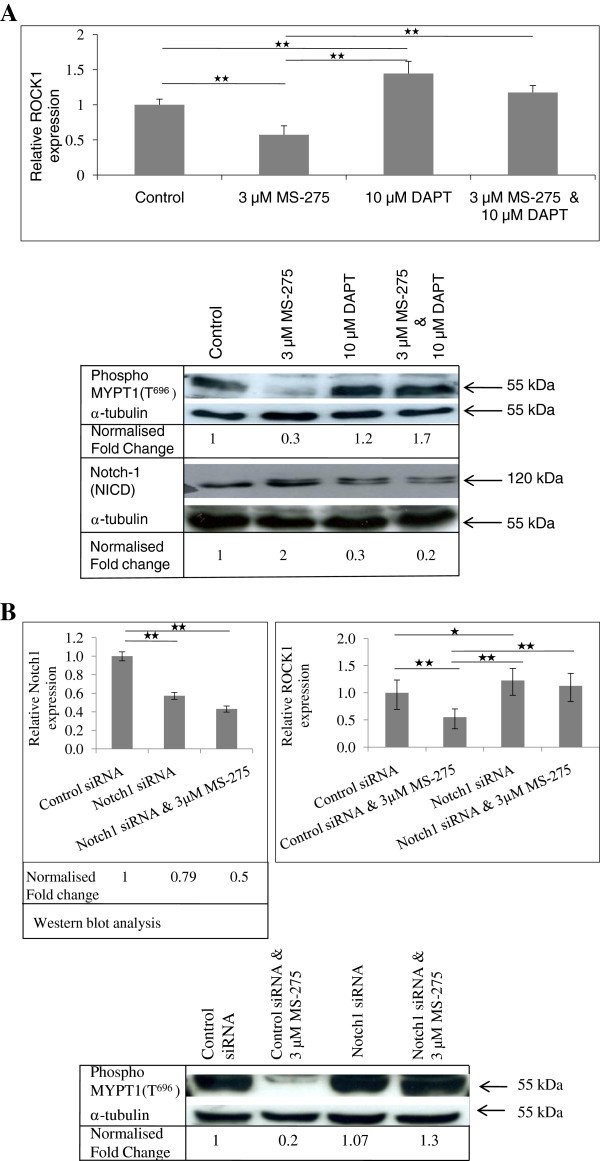
** Inhibition of Notch1 abrogates ROCK1 downregulation by MS-275 in HD matrices.A**, MTLn3 breast cancer cells invaded into HD matrix for 24 h and were treated with MS-275, the Notch1-1 inhibitor, DAPT, and combined treatments for 48 h. MS-275 alone decreased ROCK1 transcript level but when used in combination with DAPT, this effect was abrogated. Western blotting for Notch1 demonstrated that increased expression of Notch-1 intracellular domain (NICD), following MS-275 treatment. Conversely, ROCK activity was reduced by MS-275 and this effect was lost in cells that were inhibited for Notch-1. **B**, the knockdown of Notch1 by siRNA demonstrated, there was a reversal of ROCK inhibition by MS-275 to control levels in cells knockdown for Notch1. The activity of ROCK1 was also increased by 1.3 fold when cells were treated with Notch1 siRNA and MS-275 together. Ratio measurements were obtained following normalisation with α–tubulin. Bars indicate standard error from two biological replicates and the experiments were performed thrice for qPCR and twice for Western blot analysis. 1-way ANOVA followed by post-hoc Tukey’s test indicating significant difference at ** *p* < 0.01.

HD matrix reduced Notch1 expression that was reversed by MS-275. In human primary keratinocytes, adenoviral transfection of p53 suppressed the expression of ROCK1 and conversely, downregulation of p53 using siRNA upregulated the expression of ROCK1 [45]. Furthermore, knockdown of p53 downregulates Notch1 expression while p53 activation by ionising radiation or actinomycin D upregulate Notch1 in human cervical keratinocytes [46]. Yugawa et al., 2007, reported that human Notch1 has several putative p53-resposive sequences and p53 transactivate the Notch1 promoter and regulates its expression [46]. Notch1 is also regulated independently of p53, for example, in the p53 deficient cell line, T47D, activation of Notch1 occurs through Discoidin domain receptor tyrosine kinase1 (DDR1) [47]. Furthermore, it is well studied that HDAC inhibitors, for example VPA, SBHA and TSA increased Notch1 at transcript and protein level in many cancers [48,49]. Therefore, it is possible that Notch1 and/or p53 might be responsible for the indirect effect of MS-275 on ROCK1 expression. To test this, we first determined whether the gene expression of Notch1 and p53 were regulated by matrix density, and whether this was in turn affected by MS-275. HD matrix downregulated the expression of Notch1 (Figure 6B). Furthermore, MS-275 increased Notch1 transcript levels (Figure 6C). Western blot analysis of Notch1 showed that MS-275 also increased the protein levels of the intracellular domain of Notch1 (NICD), the active form of Notch1 (Figure 7A). Unlike Notch 1 expression, expression of p53 was not altered by matrix density (Figure 6B) or by MS-275 treatment ( Additional file 3: Figure S2). Therefore, Notch1 is a candidate suppressor of ROCK1 whereby its upregulation by MS-275 might be responsible for indirectly reducing ROCK1 levels. CHX had no effect on Notch1 expression suggesting a direct effect of MS-275 in elevating Notch1 levels (Figure 6C). Therefore, the expression of Notch-1 might be downregulated by histone deacetylation, leading to increased expression of ROCK1 in HD matrix.

In contrast to the effects on Notch1, CHX alone or CHX and MS-275 treatments caused significant increases in the expression of p53. There is no precedent for this observation for p53 but cell cycle genes have been shown to be upregulated by CHX [50,51]. Furthermore, synergy between EGF and CHX has led to the upregulation of actin mRNA transcript [52]. It is likely that these effects are due to blockade of an inhibitor by CHX so that in some cases, inhibition of protein synthesis can lead to upregulatory effects. This reasoning may apply to CHX upregulation of p53 via either increased stability of p53 mRNA or the transcription rate or both.

### MS-275 regulated Notch1 expression leading to the suppression of ROCK1 in HD matrices

To determine whether the increase in Notch1 expression by MS-275 might be responsible for the downregulation of ROCK1, we used the Notch1 inhibitor, DAPT, in combination with MS-275 in quantitative RT-PCR and kinase assay experiments. The Notch pathway has a critical cleavage step involving the γ-secretase complex of four proteins. Enzymatic cleavage of Notch by γ-secretase complex is essential for the formation of the active intracellular Notch domain (NICD). DAPT is a potent γ-secretase inhibitor that inhibits the formation of NICD and its downstream pathways [53]. The combination of DAPT and MS-275 abrogated the down regulation of ROCK1 by MS-275 alone (Figure 7A). The results were also confirmed using *SMART Pool* siRNA to knockdown the expression of Notch1 (Figure 7B). Similar data were found when replacing MS-275 with VPA. In HD matrix, VPA significantly suppressed ROCK1 expression whereas DAPT increased ROCK1 mRNA level. Treatment with both VPA and DAPT abrogated the effect of VPA alone, increasing ROCK1 expression to control levels ( Additional file 2: Figure S1).

## Discussion

Breast tumours have a tendency to be highly desmoplastic with high collagen content. This work explores ROCK1 activity, regulation and cell contractility function during cell migration in high-density (HD) matrices. Live-cell imaging showed that tumour cells navigated through HD matrices by contraction of the cell body. Treatment with inhibitors demonstrated a role for ROCK1 and MMPs in cell migration. There was increased expression of invasive genes in HD compared to LD matrices including ROCK1, whereby both its expression and activity were significantly upregulated in denser matrices. This effect of the microenvironment on ROCK1 was sensitive to treatment with a HDAC inhibitor, MS-275, which upregulated Notch1 that in turn, suppressed ROCK1. This was shown by downregulation of Notch1 using siRNA knockdown and DAPT, which abrogated the inhibition of ROCK1 by MS-275.

Dense breast tissue shows increased stromal collagen and analyses of tumour material indicate that cancerous breast tissues are stiffer than healthy tissue [8]. Stiffness or resistance to deformation measured from Young’s modulus of collagen matrices is dependent on the number of fibrillar cross-links and higher fibre densities [19,54,55]. Stiffer matrices promote invasion by increasing the numbers of active invadopodia [56] and increase cell proliferation by elevating Rho-GTPase activity and cell adhesion [57]. Tumour cells in turn, remodel the extracellular matrix for example, by realigning randomly organised collagen fibres into a radial configuration to help facilitate local invasion [14,15,58]. Tumour cells are also known to use protease to cleave ECM components and together with other mechanisms to contract and reorganise the collagen matrix to provide space required for cell migration [59]. It is conceivable that matrix reorganisation via pushing of protrusions, contraction of the cell body and local matrix proteolysis serve to reduce matrix stiffness and facilitate cell migration. This study showed that levels of ROCK transcript, protein and protein activity were significantly upregulated in stiff matrices coincident with the observation of cell body contractility utilised for migration. Unlike other biological programs such as proliferation and differentiation where cells are committed to specific pathways, cells can switch between regulatory pathways and migration modes for invasion. Protrusion-, contractility- or protease-led mechanisms are interchangeably utilised by tumour cells. These are dependent on environmental conditions and cell proclivities related to genetic make-up governing polarity, adhesion and cytoskeletal functions. Variations in these factors lead to a number of permutations in the migration mode of tumour cells. For example, blockade of MMPs causes mesenchymal tumour cells to switch to cell contractility for migration similar to amoeboid cells in LD matrices [15,22,57,60].

In HD matrix, ROCK inhibition had no effect on migration even though live DIC microscopy showed evidence of cell contractility. It is possible that in the absence of ROCK, protease-led migration might compensatory. Indeed, inhibiting both MMPs and ROCK, cooperatively/synergistically reduced migration levels, albeit at the lower end of the GM6001 concentration (12.5 μm) used. This suggests that at a critical level of MMPs, ROCK is required for efficient cell migration. At higher GM6001 concentrations (above 12.5 μm), addition of ROCK inhibitors has no further effects suggesting that ROCK can no longer compensate for migration. Here, we are able to glimpse into how tumour cells are inherently plastic where cells can swap between migration modes utilising ROCK1 and/or MMPs. Residual migration (when ROCK1 and proteolysis are inhibited) suggests that a third pathway is utilised, possibly one that controls protrusion-led migration. Indeed, we observe that tumour cells migrate into dense matrices utilising enlarged protrusions that interacts with collagen fibrils to gain traction ( Additional file 1: Movie S1).

Epigenetics have been shown to play a role in regulating ROCK1 expression as a function of cell adhesion, an environmental cue [34]. Cells in suspension expressed more ROCK1 compared with adherent cells and the use of an HDAC inhibitor further increased the expression of ROCK1 in suspension cultures. The function of ROCK1 was to generate cell contractility that blocked adhesion in the cells in suspension [34]. Here we explored whether epigenetics might also play a part in the regulation of ROCK1 when cells experience microenvironmental differences in matrix stiffness. ROCK1 expression and activity was significantly upregulated in the highly elastic HD matrix compared to LD matrix. Blocking HDAC function using MS-275 downregulated ROCK1 and this could result from either direct or indirect effects of the drug.

HDACs deacetylate histones reducing accessibility of DNA to the transcription machinery resulting in inactive chromatin. Furthermore, histone deacetylation can also lead to methylation-dependent transcriptional activation [61,62]. There are two possibilities as to how HDACs might increase ROCK1 transcript in HD matrix. This might occur directly through HDAC promotion of histone methylation at H3K4_Me_ and activation of ROCK1 gene transcription (as opposed to downregulating genes at other methylated sites such as H3K9_Me._). The alternative hypothesis is that in HD matrix, HDAC suppresses an inhibitor of ROCK1 so that addition of MS-275 abrogates this suppression, leading to the downregulation of ROCK1. To test this, we used cycloheximide (CHX) to block protein translation and showed that CHX prevented the downregulation of ROCK1 transcript and protein activity in the presence of MS-275. This suggests that the effect of MS-275 on ROCK1 is indirect and it is dependent on another protein(s) upregulated by MS-275.

ROCK activity is regulated by Rho GTPase, which frees the kinase region from the autoinhibitory carboxy-terminal region of ROCK1 [63] and it is also activated autonomously from Rho [64]. ROCK phosphorylates substrates that function in the assembly of actin filaments and in cell contractility including ezrin-radixin-moesin (ERM) proteins and MLC [65]. Phosphorylation of the MLC of myosin II activates myosin ATPase and consequently promotes cell contractility [63]. Furthermore, ROCK also phosphorylates the myosin-binding subunit (MYPT1) of myosin light–chain phosphatase (MLCP), a negative regulator of MLC [66], resulting in enhanced contractility. We show that using blebbistatin to block myosin II, downstream of ROCK, has no effect on cell migration ( Additional file 4: Figure S3).

Apart from self-regulation at the protein level, ROCK can be controlled at the transcript level. In keratinocytes, p53 positively regulates Notch1 and both these factors inhibit ROCK1/2 [45]. Notch is a type I transmembrane receptor with a key role in cell fate determination and the differentiation of cells during development. Inhibition of Notch increases tumour formation by primary human keratinocytes expressing oncogenic Ras, suggesting a tumour suppressor role for Notch. Blockade of Notch also suppressed differentiation and increased stem cell populations [45]. The binding of cognate ligands to the Notch receptor is followed by proteolytic cleavage of Notch, releasing its intracellular active domain. Notch translocates to the nucleus and interacts with DNA-binding proteins such as CSL, converting it from a transcriptional repressor to an activator [67,68]. Notch also binds Mastermind-like 1 (MAML1) to further elevate CSL-regulated transcriptional activation [69]. The Notch/CSL/MAML pathway targets the HES and HERP families of basic helix-loop-helix (bHLH) transcriptional repressors [70]. Conserved HES-binding sites in turn, can be found in the promoter regions of ROCK2 and MRCKα genes, the effectors of RhoA and CDC42, respectively. Notch promotes the repressor function of HES1 leading to the downregulation of ROCK2 and MRCKα gene expression [45]. Furthermore, use of DAPT increased the expression of ROCK1 and 2, supporting the idea that Notch1 normally controls these genes in keratinocytes to prevent tumour progression [45]. The transcript levels of Notch have been shown to be upregulated in mouse embryos treated with trichostatin A, a potent HDAC-inhibitor [71]. Therefore, there is evidence to suggest that Notch1 not only negatively regulates ROCK1 at the promoter level but that HDAC inhibitors upregulate Notch1 gene expression.

In HD matrix, we find that Notch1 but not p53 was upregulated by MS-275 and the increase in Notch1 levels was independent of CHX. When Notch1 activation was blocked using a γ-secretase inhibitor, DAPT, or when Notch1 levels were reduced by pooled siRNA transfection, the effect of MS-275 on ROCK1 activity was abrogated. The data suggest that MS-275 directly upregulates Notch1, which in turn blocks ROCK1 expression perhaps via repressor activities on the ROCK1 promoter [45].

## Conclusion

**Figure 8:**
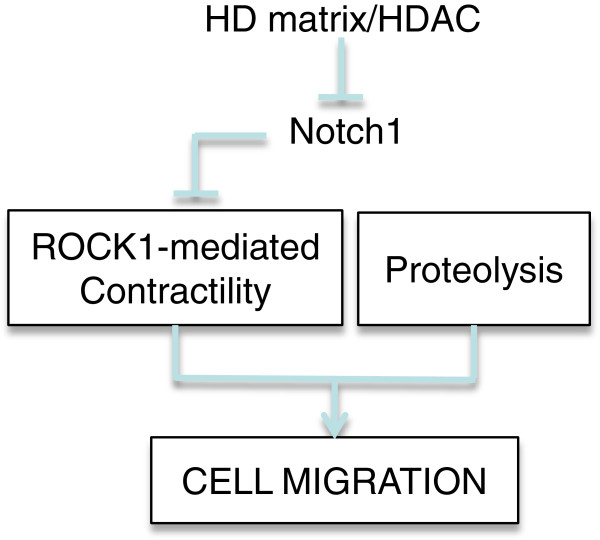
** Regulatory pathways in the regulation of ROCK1 and cell migration in HD matrix.** HD matrix inhibits Notch1 via HDAC(s) leading to upregulation of ROCK1 transcript, protein and activity levels. ROCK1 in turn increases cell contractility and together with matrix proteolysis, facilitate cell migration into HD matrix.

This work shows that amoeboid tumour cells migrate in stiff matrices by upregulating ROCK1 activity and cell contractility via an epigenetically-derived, Notch1-dependant mechanism (Figure 8). However, the requirement for ROCK1 is conditional upon the availability of other mechanisms such as proteolysis-assisted migration.

## Methods

### Reagents

N- (2-aminophenyl)-4-[N- (pyridin-3-yl -methoxycarbonyl) aminomethyl) benzamide (MS-275) was purchased from Enzo Life Sciences, International (Plymouth Meeting, PA). (*R*)-(+)-*trans*-4- (1-Aminoethyl)-N- (4-Pyridyl) cyclohexanecarboxamide dihydrochloride monohydrate (Y-27632), N- [N- (3,5-Difluorophenacetyl)-L-alanyl)-S-phenylglycine t-butyl ester (DAPT), and 3-[2-(3,5-Dimethyl-2-oxocyclohexyl)-2-hydroxyethylglutarimide, actidione, naramycin A (Cycloheximide), and Valproic acid were purchased from Sigma (Sigma-Aldrich, Corp., St. Louis, Mo). GM6001 was purchased from Calbiochem (Calbiochem, SanDiego, CA).

### Mammary tissue accrual and histochemistry

Mammary tissue containing DCIS was surgically resected in accordance with standard health care procedures and with human research ethics committee (HREC) approvals (USyd #09-2009/12168 and Peter MacCallum #08/21). Human studies were performed in accordance with the ethical standards laid down in the 1964 Declaration of Helsinki and its later amendments. Patients have given informed consent to participate in the study. Mammary tissue collected from patients undergoing subcutaneous mastectomy for breast carcinoma was sliced thickly (~1 cm thick) in the superior-inferior axis, fixed in neutral buffered formalin and subjected to specimen radiography. Areas containing HMT or LMT were removed from the slice after registration with a specimen mammogram of the slice, transferred into 9.0% sucrose in 0.1 M phosphate buffer for 12 h and embedded in OCT compound. Routine 5 μm thick sections were cut with a Bright OTF cryostat at −30°C and fixed with absolute acetone at −20°C for 10 min. Masson’s Trichrome or H&E were used to stain these sections.

### Picrosirus red collagen assay

Collagen content within HMT and LMT tissues was estimated against picrosirius red collagen standards (up to 31.93 mg/cm^3^). HMT and LMT tissues, HD matrices and samples with known collagen concentrations were stained with 200 μl 1% (w/v) Sirius red (Sigma-Aldrich) in saturated picric acid solution (Sigma-Aldrich) for 30 min. The samples were then washed in 0.02 M acetic acid followed by 10000 x g centrifugation for 1 min until all unbound stain was removed. Collagen-bound stain was then extracted in the presence of 0.5 M NaOH. Absorbance of the eluted stain was read at 531 nm using a VictorX Multilabel plate reader (PerkinElmer, Waltham, MA).

### Electron microscopy

Samples were fixed in 2.5% glutaraldehyde followed by 1% Osmium tetroxide, dehydrated in a graded series of ethanol and critical point dried. SEM images were taken with the Zeiss Ultra field emission scanning electron microscopy (FESEM) (Carl Zeiss), using the SE2 or In Lens detector, 2 kV and of a working distance of 4 mm.

### Cell culture and transfection

Rat mammary adenocarcinoma cells, MTLn3, were grown in Advanced Dulbecco’s modified Eagle’s medium (Adv D-MEM), supplemented with 5% fetal bovine serum, 1% GlutaMax and 1% antibiotic- antimycotic (Invitrogen/Gibco, Carlsbad, CA), at 37°C and 5% CO_2_ incubator and subcultured at 80% confluency. Twenty-four hours prior to transfection, 4 × 10^4^ cells were seeded on 12 well plates. Rat Notch-1 ON-TARGET plus *SMART Pool* siRNA from Dharmacon (Thermo Fisher Scientific, Lafayette, CO) and non-targeted scrambled siRNA from Ambion (Ambion, Austin, TX) were transfected using DharmFECT 1 transfection agent. Twenty-four hours after transfection, cells were trypsinized and grown on the high-density collagen matrices for 24 h and then fresh medium was replaced with or without drugs. Forty-eight hours after drug treatments, cells were harvested and total RNA and protein were extracted for further analysis.

### Preparation of collagen matrices and seeding cells

High-density collagen matrices were prepared by using 20 mg/cm^3^ concentrated collagen. Around 500 μl of 10 mg/cm^3^ acid-solubilised monomeric rat tail type I collagen (BD Biosciences, San Jose, CA) was centrifuged in a microcon-YM-30 column containing a 30 kDa filter cartridge (Millipore, Billerica, MA) for 60 min. Around 50–100 μl of concentrated collagen was added to the 14 mm microwell of a glass-bottom culture dish (MatTek Corporation, Ashland, MA). Collagen was polymerised by NH_4_OH vapour and the matrices were washed with 1X PBS and incubated in complete medium overnight in a humidified, 37°C and 5% CO_2_ incubator. Low-density (LD) collagen matrices were made by appropriate dilution of collagen followed by polymerisation at 37°C incubator. The present model studies how tumour cells enter high-density (HD) collagen matrices that are similar in density to stromal matrices. MTLn3 cells (10 X 10^4^) were seeded on top of control LD and HD collagen matrices and were allowed to migrate for 24 h. The samples were then washed to remove non-adherent cells, treated with drugs or vehicle for another 48 h (unless otherwise stated) and then prepared for imaging, total RNA extraction or protein extraction. At the end of the 72 h in control untreated samples, 89% (n = 4 experiments) and 100% (n = 2 experiments) of total cells in the matrices have invaded into LD and HD matrices, respectively.

### Light microscopy and cell invasion quantitation

Differential interference contrast (DIC) live cell imaging was performed on an Olympus CellR Live-Cell Microscope equipped with a 5% CO2 and 37°C enclosure (Olympus). Images of Masson’s Trichrome were captured using the Zeiss Axioskop 2 MAT Light Microscope (Carl Zeiss, Goettingen, Germany).

For quantitation of migration depth, treated and control cells were washed in pre-warmed PBS and cells were fixed in 4% paraformaldehyde for 20 min, followed by washing, and permeabilisation in 0.1% Triton X-100 for 5 min. Cells were stained in with Alexa fluor 488 phalloidin (Invitrogen) for 15 min. Stained cells were imaged by confocal and collagen by reflection microscopy using an Olympus FluoView FV1000 microscope (Olympus, Tokyo, Japan). Reflection microscopy was performed by scanning with either the 488 or 559 nm laser line and detected by passing the reflected light through the DM405/488/559/635 dichroic and capturing within the bandwidth of 485–490 nm for the 488 scan or 555–560 nm for the 559 nm scan. Individual stacks were projected along the z-axis and migration depth was measured from the collagen surface to the ventral cell surface using ImageJ. Cells were sampled randomly for imaging and for the analyses.

### Extraction of total RNA

Total RNA was extracted from treated and control cells to study the gene expression pattern. Culture medium was aspirated and cells were washed in pre-warmed PBS. Collagen matrices were transferred into 1.5 ml of eppendorf tubes and 1 ml of TRIzol® reagent (Invitrogen) was added to each gel and homogenized by pipetting up and down. After homogenisation, the tubes were incubated for 5 min at room temperature and 0.2 ml of chloroform was added to each tube and shook for 15 s. The mixture was incubated for another 5 min at room temperature and spun down at 13,000 rpm at 4°C for 15 min. The upper aqueous layer was transferred into a new tube and same volume of 70% ethanol was added into the tubes. It was mixed thoroughly and mixture was transferred into the RNeasy Mini spin column (Qiagen, Hilden, Germany) and remaining steps were followed according to manufacturer’s protocol.

### Reverse transcription and real-time quantitative PCR (RT-qPCR)

Reverse transcription was performed using SuperScript III First-Strand synthesis system (Invitrogen) according to the manufacturer’s protocol. We have quantified expression levels of some of the genes, which are responsible for cell invasion and proliferation using fluorescence detection method (primer list attached on Additional file 5: Table S1); 10 μl of Brilliant SYBR master mix (Stratagene, La Jolla, CA) or Express SYBR® GreenER (Invitrogen), 0.25 μM primers and 1 μl of cDNA were used in 20 μl of total reaction. The relative mRNA level was calculated by the 2^-ΔΔCT^ method. GAPDH was used as an internal control. RT-qPCR experiments were performed in Rotagene 6000 from Corbett Research. Initial denaturation was at 95°C for 10 min. The cycling conditions afterwards were as follows: 95°C for 10 s, annealing dependent on the primer temperature, and elongation at 72°C for 20–30 s. Melting curve analysis was performed between 72°C and 98°C. Each sample was assayed in triplicate, each experiment had at least 2 biological replicates, and each experiment was performed at least 3 times.

### Protein extraction and western blot analysis

Collagen matrices containing migratory cells were washed in PBS and transferred to 24 well plates. Matrices were digested by 0.5 mg/ml of collagenase (Sigma-Aldrich) in Kreb’s Ringer buffer supplemented with 50 mM CaCl_2_ at 37°C for 30 min. Cells were pelleted at 2000 rpm, were washed in ice-cold PBS. RIPA buffer (Sigma-Aldrich) that contain freshly added protease inhibitor cocktail (Sigma-Aldrich), was added to each pellet, mixed thoroughly and incubated for 1 h in the ice. Cell lysate was centrifuged at 13,000 rpm for 15 min at 4°C. Concentration of protein in the supernatant was determined using Bio-Rad protein assay dye reagent (Bio-Rad Laboratories, Hercules, CA). Twenty microgram of protein was solubilised in SDS-sample buffer at 95°C for 5 min, and separated by SDS-PAGE using 8–10% resolving gels. Proteins were electroblotted onto immunoblot PVDF membrane (Millipore). After transfer, membranes were blocked in 5% skim milk/TBST for 1 h and the membrane washed three times in TBST. The membranes were incubated overnight at 4°C in 1% skim milk/TBST containing primary antibodies that were specific for ROCK1 (H-85) (1:500) or Notch1 (1:1000) from Santa Cruz (Santa Cruz Biotechnology, SantaCruz, CA). After washing with TBST, membranes were incubated with 1% skim milk/TBST containing secondary antibody conjugated to horseradish peroxidase (GE Healthcare Bio-Sciences Corp, Piscataway, NJ) for 1 h. The blot signal was visualized by using enhanced chemiluminescence hyperfilm (GE Healthcare). Blots were then stripped at room temperature for 30 min in Restore plus western blot stripping buffer (Thermo Fisher Scientific) and reprobed with mouse anti-α-tubulin (1:2500) monoclonal antibody.

### ROCK activity assay and immunoblotting

ROCK activity was measured using ROCK activity immunoblot kit from Cell Biolabs Inc (Cell Biolabs Inc. San Diego, CA). The reaction was carried out with slight modification of manufacturer’s protocol; 65 μl of Kinase/ATP/Substrate solution was prepared by adding 3 μl of 10 mM ATP and 3 μl of ROCK substrate (recombinant MYPT1) to 59 μl of freshly prepared 1X Kinase buffer and then 10 μl of 1X Kinase/ATP/Substrate solution and 10 μg of whole cell lysate protein from treated or untreated cells were used for the kinase reaction. The kinase reaction was initiated by incubating tubes at 30°C for 1 h with gentle agitation and stopped by adding SDS-sample buffer. Protein mixture was boiled at 95°C for 5 min. Protein was separated in 12% resolving gel. Rabbit anti-phospho-MYPT1 (Thr^696^) primary antibody (1:1000) was used to detect the phosphorylated MYPT1 substrate.

### Rheology

Rheological analyses for measuring the viscoelastic properties of collagen gels were performed using the Physica MCR 301 (Anton-Paar GmbH, Austria) and a cone plate of 50 mm in diameter. Collagen gels (50 mm in diameter and 1 mm in thickness) were loaded onto the rheometer lower plate. The upper cone plate was slowly lowered onto the collagen gel until full contact was achieved. Frequency sweep oscillations from 0.01 to 30 Hz were performed and the storage modulus (G’) and loss modulus (G”) were recorded. For the frequency sweep oscillation measurements, 1% maximal strain and shear rates from 0.000626 to 1.87 1/s was used.

### Morphometric measurements and statistics

Cell morphology was analysed using ImageJ. The outline for each cell was traced and parameter measurements were obtained for Circularity and Aspect Ratio. The data were transferred to Microsoft Excel for analysis and statistical evaluations. Data were expressed as Mean ± SD. Analyses were performed by Student *t*-test or one- way ANOVA followed by post-hoc Tukey’s test. P values less than or equal to 0.05 were considered statistically significant.

## Abbreviations

3D, Three -dimensional; ECM, Extra cellular matrix; HD, High-density; HDAC, Histone deacetylase; LD, Low-density; NICD, Intracellular domain of Notch1.

## Competing interests

The authors declare that they have no competing interest.

## Author’s contribution

VR designed the experiments, analysed gene expression profiles, performed siRNA knockdown experiments, Q-PCR and western analyses, ROCK inhibitor assays and contributed to the writing. SF analysed the concentration, organization and biophysical properties of collagen in HMT, LMT and HD matrix, developed HD matrices, and performed live-cell imaging of cell migration in the presence and absence of ROCK inhibitors. JZ performed and analysed the data for ROCK migration assays and participated in manuscript writing. HC performed the ROCK and MMP inhibitor studies. JGL assisted in the development of HD matrix and provided intellectual input on collagen gel properties. ET provided the clinical samples and intellectual input oriented towards clinical assessment of dense tissue and MMP-related cell processes and proofread the manuscript. LS provided the intellectual direction, coordinated the experiments and wrote most of the manuscript. All authors read and approved the final manuscript.

## Supplementary Material

Additional file 1 **Movie S1.** Tumour cell migration in HD matrix by live cell imaging. Live cell imaging from differential interference contrast (DIC) microscopy showing a tumour cell (MTLn3) moving through HD matrix. Frame rate = 15 s/frame. Bar = 10 μm. Click here for file

Additional file 2 **Figure S1.** Effects of blebbistatin on cell migration in HD matrix. Blebbistatin (6.25 uM) was added 5 h after seeding MTLn3 cells onto HD matrix and the cells were allowed to migrate for a further 24 h prior to fixation, staining with phalloidin actin, imaging and measurements of invasion depth. Graph illustrates the degree of migration in microns of vehicle- and blebbistatin-treated cells. Click here for file

Additional file 3 **Figure S2. **HDAC inhibitor, VPA suppresses the ROCK1 expression. MTLn3 breast cancer cells were allowed to invade into HD matrix for 24 h, were treated with VPA, DAPT, and combined treatments for 48 h. The cells were harvested for mRNA expression analysis. Q-PCR results showed that VPA alone decreased ROCK1 transcript level (40%) compared with control. Conversely, blocking of Notch-1 increased the ROCK1 mRNA level (75%) compared with control. Combination of VPA with DAPT, brought back the transcript level of ROCK1 similar to control. VPA increased the expression of Notch-1 transcript (150%) compared with control. DNA gel pictures represent the Q-PCR results. Relative expression was calculated using DDCt method and GAPDH used as internal control. Bars indicate standard error from two biological replicates and the experiments were repeated at least twice. 1-way ANOVA followed by post-hoc Tukey’s test indicating significant difference at * p < 0.05 and** p < 0.01. Click here for file

Additional file 4 **Figure S3.** CHX increases p53 expression in HD matrix. MTLn3 breast cancer cells were allowed to invade into HD matrices for 24 h before treatment with 1 μM and 3 μM MS-275, 10 μg/ml of cycloheximide (CHX) and both CHX and MS-275 and cells were grown for further 24 h. Treatment of MS-275 at both concentrations did not affect the expression of p53 transcript in HD matrix. However, treatment of CHX increases the p53 expression. Bar indicates standard error from two biological samples and experiments were performed two times. 1-way ANOVA followed by post-hoc Tukey’s test indicating significant difference at either * p < 0.05 or ** p < 0.01. Click here for file

Additional file 5 **Table S1.** Primers used for real time RT-PCR reactions. The primers were designed using Primer3 software. All the primer pairs spanned an exon-exon junction to prevent genomic DNA amplifications. The specificity of all primer sequences were tested with Basic Local Alignment Search Tool (BLAST; http://www.ncbi.nlm.nih.gov/blast/Blast.cgi). The annealing temperature of the primers was ~ 60oC and size of the PCR amplicons were range from 200–250 bp. Click here for file
