# Supplementary figures and images for: Regulation of ROCK1 via Notch1 during breast cancer cell migration into dense matrices
Source: BMC Cell Biol. 2012 May 14;13:12. doi: 10.1186/1471-2121-13-12 (PMC3520698; doi:10.1186/1471-2121-13-12)

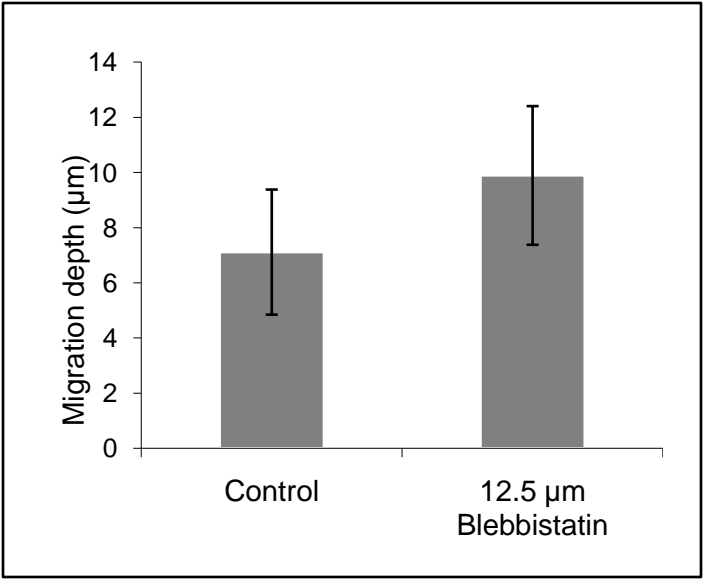

Supplementary Figure 1

Supplement: Additional file 2 — Figure S1. Effects of blebbistatin on cell migration in HD matrix. Blebbistatin (6.25 uM) was added 5 h after seeding MTLn3 cells onto HD matrix and the cells were allowed to migrate for a further 24 h prior to fixation, staining with phalloidin actin, imaging and measurements of invasion depth. Graph illustrates the degree of migration in microns of vehicle- and blebbistatin-treated cells. [file 1471-2121-13-12-S2.pdf]

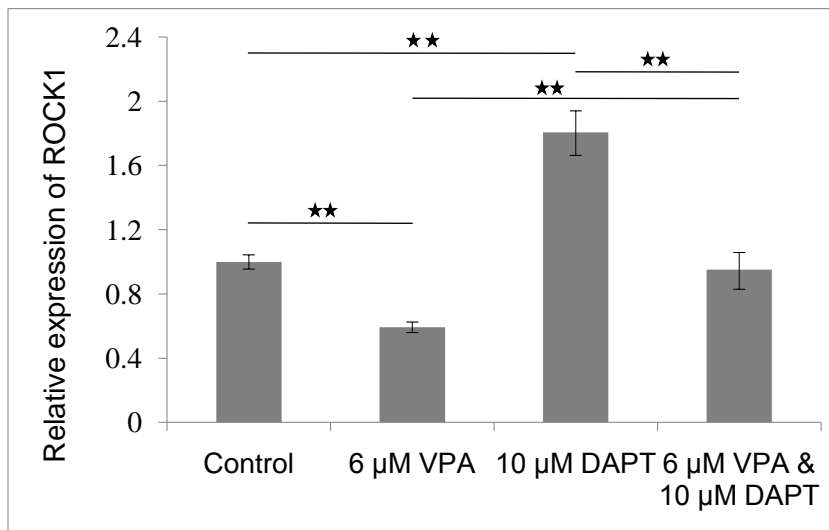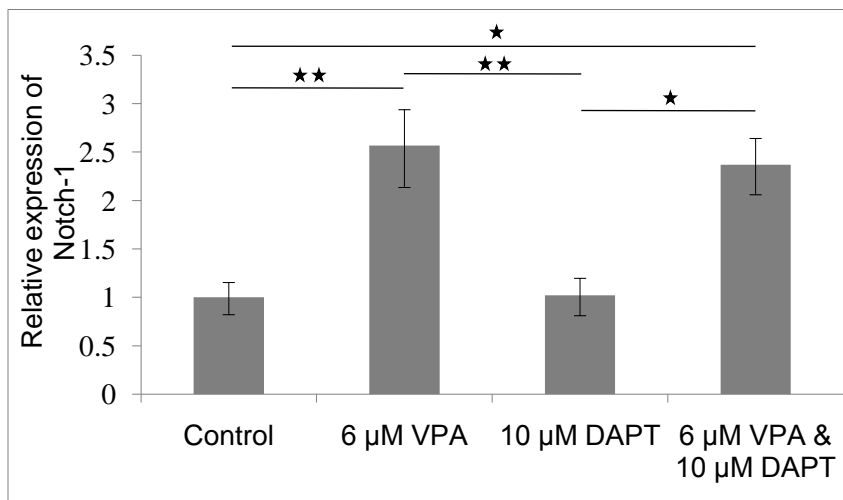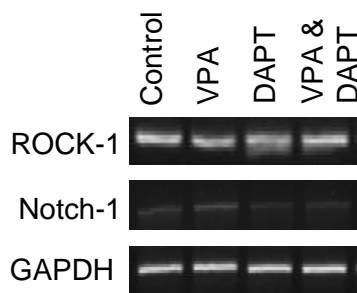

Supplementary Figure 2

Supplement: Additional file 3 — Figure S2. HDAC inhibitor, VPA suppresses the ROCK1 expression. MTLn3 breast cancer cells were allowed to invade into HD matrix for 24 h, were treated with VPA, DAPT, and combined treatments for 48 h. The cells were harvested for mRNA expression analysis. Q-PCR results showed that VPA alone decreased ROCK1 transcript level (40%) compared with control. Conversely, blocking of Notch-1 increased the ROCK1 mRNA level (75%) compared with control. Combination of VPA with DAPT, brought back the transcript level of ROCK1 similar to control. VPA increased the expression of Notch-1 transcript (150%) compared with control. DNA gel pictures represent the Q-PCR results. Relative expression was calculated using DDCt method and GAPDH used as internal control. Bars indicate standard error from two biological replicates and the experiments were repeated at least twice. 1-way ANOVA followed by post-hoc Tukey’s test indicating significant difference at * p < 0.05 and** p < 0.01. [file 1471-2121-13-12-S3.pdf]

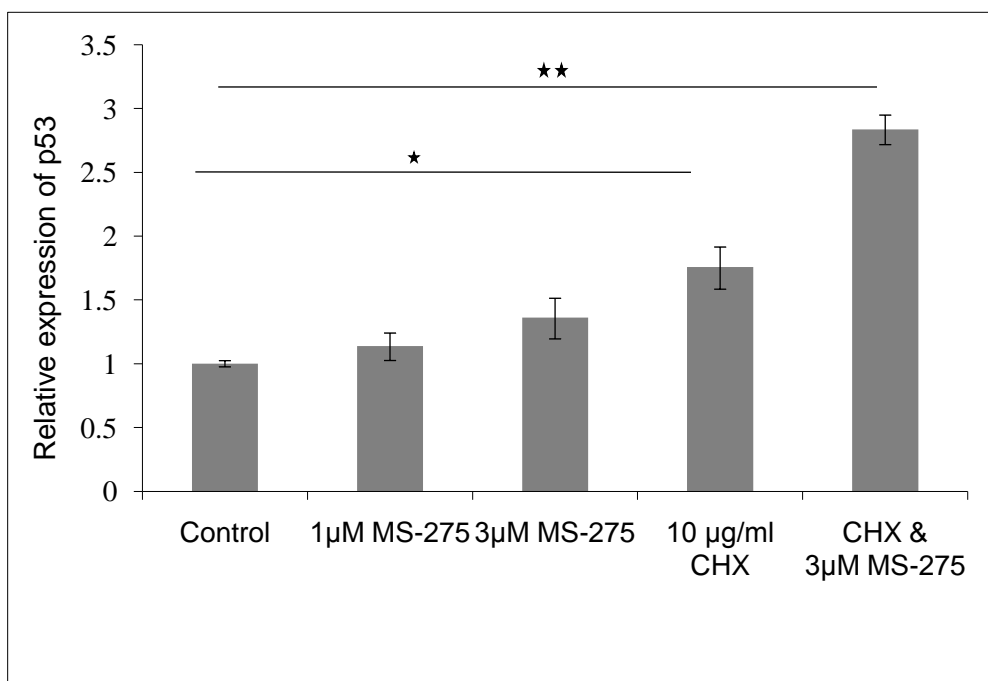

Supplementary Figure 3

Supplement: Additional file 4 — Figure S3. CHX increases p53 expression in HD matrix. MTLn3 breast cancer cells were allowed to invade into HD matrices for 24 h before treatment with 1 μM and 3 μM MS-275, 10 μg/ml of cycloheximide (CHX) and both CHX and MS-275 and cells were grown for further 24 h. Treatment of MS-275 at both concentrations did not affect the expression of p53 transcript in HD matrix. However, treatment of CHX increases the p53 expression. Bar indicates standard error from two biological samples and experiments were performed two times. 1-way ANOVA followed by post-hoc Tukey’s test indicating significant difference at either * p < 0.05 or ** p < 0.01. [file 1471-2121-13-12-S4.pdf]
